# Association between gastroesophageal reflux disease and coronary atherosclerosis

**DOI:** 10.1371/journal.pone.0267053

**Published:** 2022-05-20

**Authors:** Ji Hyun Song, Young Sun Kim, Su-Yeon Choi, Sun Young Yang

**Affiliations:** Department of Internal Medicine, Healthcare Research Institute, Seoul National University Hospital Healthcare System Gangnam Center, Seoul, Korea; University of Kentucky, UNITED STATES

## Abstract

**Background and aim:**

Gastroesophageal reflux disease (GERD) typically presents with symptoms of heartburn and acid regurgitation but occasionally manifests as atypical chest pain. Coronary artery disease (CAD) and GERD share some risk factors, such as smoking and obesity. The aims of this study were to evaluate the association between GERD and coronary atherosclerosis and to assess the risk factors for coronary atherosclerosis in GERD patients.

**Methods:**

A total of 16616 subjects who underwent upper gastrointestinal endoscopy from 2003 to 2017 and a cardiac computed tomography (CT) scan within one year were included in this study. Coronary atherosclerosis was evaluated by the coronary artery calcium score (CACS). The severity of GERD was evaluated based on endoscopic findings using the Los Angeles classification.

**Results:**

The proportion of high CACSs (≥100) increased significantly in subjects with severe GERD (*p* = 0.008). However, the presence of a high CACS did not increase the risk of GERD (OR = 1.007, 95% CI 0.857–1.182), nor did that of GERD increase the risk of a high CACS (OR = 1.018, 95% CI 0.865–1.198). The risk factors for a high CACS in GERD patients included age (OR = 1.087, 95% CI 1.066–1.109), male sex (OR = 5.645, 95% CI 2.561–12.446), hypertension (OR = 1.800, 95% CI 1.325–2.446), and hypercholesterolemia (OR = 1.684, 95% CI 1.213–2.338).

**Conclusions:**

Although the presence of a high CACS did not increase the risk of GERD or vice versa, the proportion of high CACSs was significantly higher in subjects with severe GERD. Therefore, it might be helpful to assess the CACS in GERD patients with multiple risk factors.

## Introduction

Gastroesophageal reflux disease (GERD) causes symptoms due to acid reflux. It typically presents with heartburn and acid regurgitation [1]. A previous study reported prevalence rates of upper gastrointestinal symptoms, including heartburn and acid regurgitation, of 22% and 16%, respectively, in the population of the United States [2]. GERD occasionally manifests as atypical symptoms, such as epigastric pain or chest pain [3]. Atypical chest pain can be seen not only in GERD but also in coronary artery disease (CAD), pulmonary disease, and panic disorders, and it is occasionally difficult to discriminate between these conditions.

CAD and GERD are known to share some risk factors, such as smoking and obesity [4]. The aims of this study were to evaluate the association between GERD and coronary atherosclerosis and to assess the risk factors for coronary atherosclerosis in GERD patients.

## Materials and methods

Between 2003 and 2017, subjects who underwent an upper gastrointestinal endoscopy and a cardiac computed tomography (CT) scan on the same day (or within 1 year for both tests) during a routine health check-up were included in this study. Most of them were asymptomatic or had only mild symptoms. After excluding patients who underwent a total gastrectomy or an esophagectomy, a total of 16616 subjects were finally enrolled. All subjects were requested to complete a questionnaire about medication, smoking history, alcohol consumption, and family history of CAD. Alcohol consumption was evaluated by ascertaining the number of drinks per week, which was defined as beer (200 ml per glass), soju (50 ml per glass), wine (120 ml per glass), or liquor (30 ml per shot), with each equivalent to approximately 10 g of alcohol per drink. The body mass index (BMI) was calculated by dividing the subjects’ measured weight (kg) by their height squared (m^2^) and used to categorize the subjects into normal (< 23.0 kg/m^2^), overweight (23.0–24.9 kg/m^2^), and obese groups (≥ 25.0 kg/m^2^) [5]. We also measured the blood pressure (BP) and laboratory values, such as the fasting blood sugar (FBS) and total cholesterol (TC) levels. Hypertension was defined as a systolic BP ≥ 140 mmHg, a diastolic BP ≥ 90 mmHg, or if the patient was taking an antihypertensive medication. Diabetes mellitus (DM) was defined as an FBS level of ≥ 126 mg/dl or if the patient was taking diabetes medication. Hypercholesterolemia was defined as present when the TC level was ≥ 240 mg/dl or when the patient was taking lipid-lowering agents.

The study protocol was approved by the ethics committee of the Seoul National University Hospital (Institutional Review Board Number: H-1703-078-839) and was conducted in accordance with the Declaration of Helsinki.

### Endoscopic diagnosis

The severity of the GERD was evaluated by the endoscopic findings using the Los Angeles (LA) classification [6, 7]. When the esophagogastric (EG) junction was observed, the presence of one or more mucosal breaks (≤5 mm) confined to the mucosal fold was classified as grade A. One or more mucosal breaks (>5 mm) that did not extend between the tops of two mucosal folds were classified as grade B. One or more mucosal breaks that were continuous between the tops of two or more mucosal folds but involved <75% of the circumference were classified as grade C, and those that involved ≥75% of the circumference were classified as grade D. Cases in which only findings, such as Z-line blurring, focal hyperemic changes, healed erosions, and whitish mucosal thickenings that were observed without a mucosal break at the EG junction, were classified as minimal change lesions (MCLs) [8, 9]. MCLs are not included in erosive reflux disease according to the LA classification system, but they are considered an early endoscopic finding of GERD and were included in this study analysis.

### Coronary artery calcium score (CACS) measurement

To evaluate the coronary atherosclerosis, the CACS extracted from the cardiac CT was used. All CT scans were performed using a 16-row multi-slice CT scanner (Sensation 16; Siemens Medical Systems) and a 256-slice scanner system (Brilliance iCT 256, Philips). A standard scanning protocol was applied with 128 × 0.625-mm section collimation, 0.27-millisecond rotation time, 120-kV tube voltage, and 800-mA tube current for the 256-slice multi-detector CT; and with a tube voltage of 120 kV, 170 effective mA, and 0.37-millisecond rotation time for the 16-slice CT. All scans were performed with electrocardiogram-gated dose modulation and data were reconstructed to generate 3-mm thick slices with a 400- millisecond acquisition window. The CACS was subsequently calculated according to the methods described by Agatston et al. [10], and using a software program (Rapidia 2.8; INFINITT). Then, subjects were classified into two groups, consisting of either a CACS of <100 or a CACS of ≥100 (high CACS) [11–13].

### Statistical analysis

Descriptive analyses included the calculations of the means and standard deviations (SD) for the continuous data and proportions for the categorical data. The Kruskal-Wallis test was used to determine whether the proportion of CACSs varied by GERD severity.

Associations between the risk factors for GERD and a high CACS were evaluated by using a logistic regression analysis to obtain the odds ratio (OR) and the corresponding 95% confidence interval (CI). The results were considered statistically significant if the two-sided *p*-value was <0.05 or if the 95% CI did not include unity. Data analysis was performed using SPSS version 25 (IBM Corp. ^©^).

## Results

**Fig 1:**
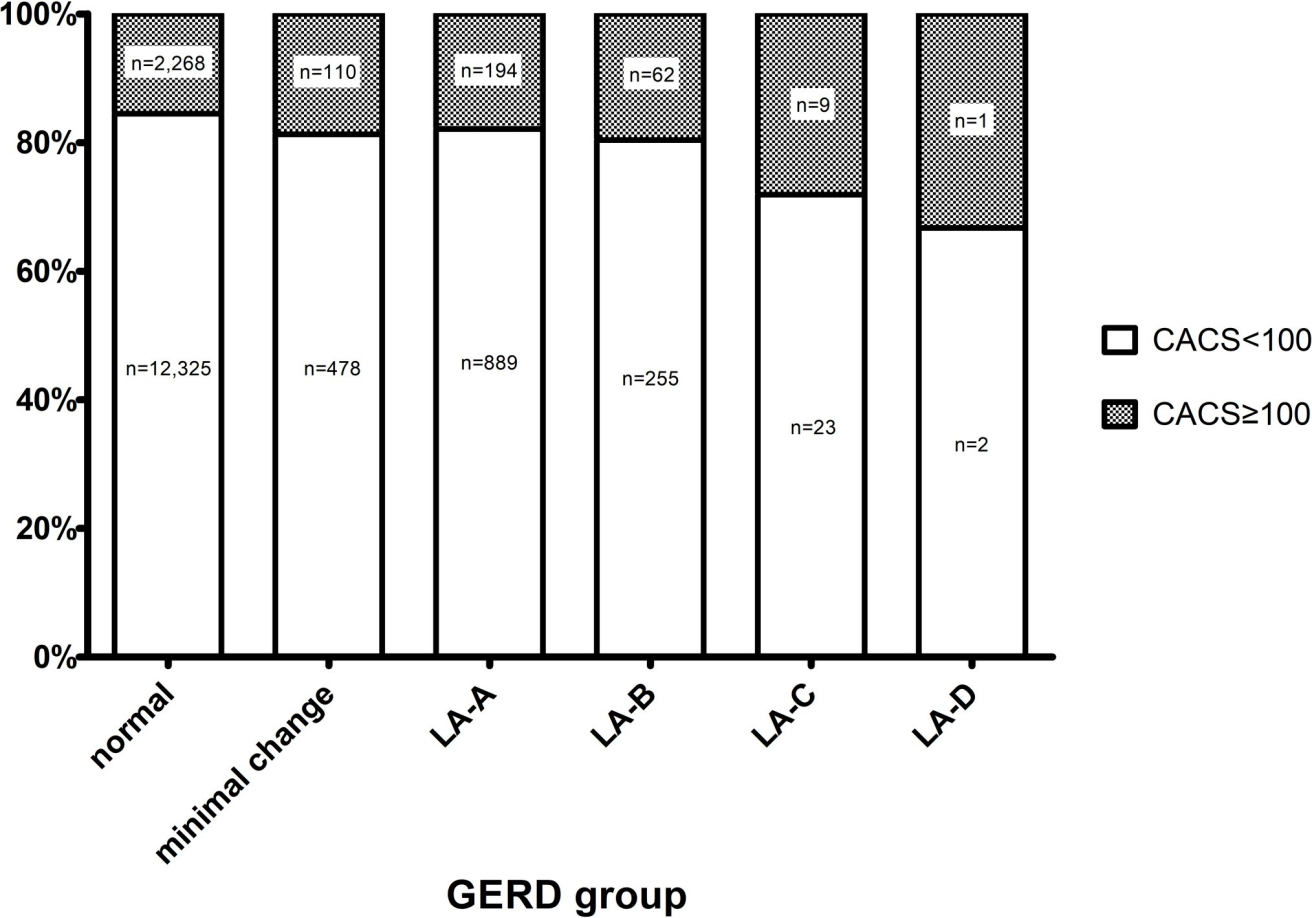
Coronary artery calcium scores (CACS) according to the severity of gastroesophageal reflux disease (GERD). The prevalence of high CACS (≥100) was increased significantly as the severity of GERD increased (*p* = 0.008).

The mean age was 55.6 years, and males represented 71% of the subjects. A total of 2641 subjects (16%) had a high CACS with a value greater than 100. GERD was diagnosed in 22% of the subjects (n = 2047). The proportion of high CACS increased as the severity of GERD increased (*p* = 0.008) (Fig 1).

The risk factors for GERD were male sex (OR = 2.302, 95% CI 1.840–2.879), current smoker (OR = 1.369, 95% CI 1.140–1.643), high BMI (overweight; OR = 1.277, 95% CI 1.083–1.506, obesity; OR = 1.534, 95% CI 1.314–1.791), and hypertension (OR = 1.181, 95% CI 1.047–1.333) (Table 1).

**Table 1:** Risk factors for GERD.

	Control (n = 14593)	GERD (n = 2023)	OR^a^	95% CI	OR^b^	95% CI
Age (years)	55.67±8.29	54.89±8.83	0.990	0.984–0.995	0.996	0.988–1.004
Male Sex	10019 (68.7)	1751 (86.6)	2.961	2.594–3.380	2.302	1.840–2.879
Alcohol (g/d)	14.33±23.34	19.36±25.43	1.007	1.005–1.009	1.002	1.000–1.004
Smoking status						
Ex-smoker	4377 (39.6)	662 (45.3)	1.782	1.560–2.034	1.114	0.942–1.317
Current smoker	2249 (20.4)	421 (28.8)	2.194	1.893–2.542	1.369	1.140–1.643
BMI(kg/m^2^)	24.24±2.90	25.14±2.88				
Overweight	4153 (29.1)	586 (29.7)	1.685	1.475–1.926	1.277	1.083–1.506
Obesity	5384 (37.7)	992 (50.2)	2.204	1.951–2.491	1.534	1.314–1.791
High CACS (**≥**100)	2268 (15.5)	376 (18.6)	1.241	1.100–1.400	1.007	0.857–1.182
Hypertension	5112 (35.0)	785 (38.8)	1.173	1.066–1.290	1.181	1.047–1.333
DM	1842 (12.6)	307 (15.2)	1.236	1.085–1.407	0.983	0.833–1.159
Hypercholesterolemia	3163 (21.7)	444 (21.9)	1.016	0.908–1.137	1.086	0.950–1.242

Data represent means ± standard deviations for continuous variables and numbers (%) for categorical variables

Abbreviations: GERD, gastroesophageal reflux disease; BMI, body mass index; CACS, coronary artery calcium score; DM, diabetes mellitus

^a^ Univariate analyses of risk factors for GERD by logistic regression.

^b^ Adjusted for age, sex, alcohol, smoking status, BMI, CACS, hypertension, DM, and hypercholesterolemia.

Table 2 shows the risk factors for a higher CACS (≥100). Age (OR = 1.107, 95% CI 1.099–1.115), male sex (OR = 3.188, 95% CI 2.599–3.911), current smoker (OR = 1.376, 95% CI 1.148–1.650), obesity (OR = 1.206, 95% CI 1.046–1.391), a family history of CAD (OR = 1.326, 95% CI 1.126–1.563), hypertension (OR = 1.977, 95% CI 1.765–2.214), DM (OR = 1.758, 95% CI 1.536–2.012), and hypercholesterolemia (OR = 1.509, 95% CI 1.336–1.705) were associated with an increased risk of a high CACS.

**Table 2:** Risk factors for high CACS (≥100).

	CACS (<100)	CACS (≥100)	OR^a^	95% CI	OR^b^	95% CI
Age (years)	54.57±7.96	60.87±8.40	1.098	1.092–1.104	1.107	1.099–1.115
Male Sex	9501 (68.0)	2269 (85.8)	2.847	2.538–3.194	3.188	2.599–3.911
Alcohol (g/d)	14.71±23.74	16.06±23.12	1.002	1.000–1.004	1.002	1.000–1.004
Smoking status						
Ex-smoker	4019(38.2)	1020 (51.4)	2.033	1.815–2.277	1.126	0.963–1.316
Current smoker	2238 (21.3)	432 (21.8)	1.546	1.348–1.773	1.376	1.148–1.650
BMI(kg/m^2^)	24.25±2.91	24.88±2.82				
Overweight	3951 (28.9)	788 (30.6)	1.508	1.345–1.691	1.106	0.952–1.285
Obesity	5185 (37.9)	1191 (46.2)	1.737	1.562–1.931	1.206	1.046–1.391
FHx of CAD	1534 (11.0)	313 (11.8)	1.089	0.957–1.239	1.326	1.126–1.563
Hypertension	4512 (32.3)	1385 (52.4)	2.306	2.120–2.509	1.977	1.765–2.214
DM	1508 (10.8)	641 (24.2)	2.645	2.384–2.934	1.758	1.536–2.012
Hypercholesterolemia	2904 (20.8)	703 (26.6)	1.380	1.255–1.519	1.509	1.336–1.705
GERD	1647 (11.8)	376 (14.2)	1.241	1.100–1.400	1.018	0.865–1.198

Data represent means ± standard deviations for continuous variables and numbers (%) for categorical variables

Abbreviations: CACS, coronary artery calcium score; BMI, body mass index; FHx of CAD, family history of coronary artery disease; DM, diabetes mellitus; GERD, gastroesophageal reflux disease

^a^ Univariate analyses of risk factors for high CACS (≥100) by logistic regression.

^b^ Adjusted for age, sex, alcohol, smoking status, BMI, family history of CAD, hypertension, DM, hypercholesterolemia, and GERD.

We evaluated the risk factors for a high CACS (≥100) in GERD patients (Table 3). Age (OR = 1.087, 95% CI 1.066–1.109), male sex (OR = 5.645, 95% CI 2.561–12.446), hypertension (OR = 1.800, 95% CI 1.325–2.446), and hypercholesterolemia (OR = 1.684, 95% CI 1.213–2.338) were associated with an increased risk of a high CACS in GERD patients. However, the smoking status, high BMI, a family history of CAD, and DM were statistically nonsignificant factors.

**Table 3:** Risk factors for high CACS (≥100) in GERD patients.

	OR^a^	95% CI	OR^b^	95% CI
Age (years)	1.081	1.066–1.096	1.087	1.066–1.109
Male Sex	3.215	2.010–5.145	5.645	2.561–12.446
Alcohol (g/d)	0.997	0.991–1.002	0.999	0.993–1.006
Smoking status				
Ex-smoker	1.368	0.977–1.915	0.905	0.593–1.381
Current smoker	1.149	0.790–1.671	1.064	0.667–1.697
BMI(kg/m^2^)				
Overweight	1.224	0.882–1.699	1.194	0.775–1.841
Obesity	1.039	0.765–1.412	0.974	0.643–1.475
FHx of CAD	0.943	0.654–1.360	1.077	0.681–1.702
Hypertension	1.713	1.367–2.146	1.800	1.325–2.446
DM	2.033	1.541–2.682	1.309	0.893–1.918
Hypercholesterolemia	1.851	1.386–2.472	1.684	1.213–2.338

Abbreviations: CACS, coronary artery calcium score; GERD, gastroesophageal reflux disease; BMI, body mass index; FHx of CAD, family history of coronary artery disease; DM, diabetes mellitus

^a^ Univariate analyses of risk factors for high CACS (≥100) in GERD patients by logistic regression.

^b^ Adjusted for age, sex, alcohol, smoking status, BMI, family history of CAD, hypertension, DM, and hypercholesterolemia.

## Discussion

The aim of this study was to evaluate the association between GERD and coronary atherosclerosis. GERD often manifests as atypical chest pain rather than typical symptoms, such as heartburn or acid regurgitation, and is often mistaken for ischemic heart disease because the symptoms are similar. Therefore, there have been many studies on GERD and coronary heart disease (CHD) [14–21]. This combined pathology can be explained by the following mechanisms. Because the esophagus has a shared innervation with the heart, GERD and CHD can similarly present with chest pain [22]. Vagal innervation is the underlying mechanism for the cardiac arrhythmias and ischemia caused by an esophageal irritation with acid reflux, as well as the esophageal spasm caused by cardiac ischemia [23]. Another mechanism, namely, endothelial dysfunction, can be considered, representing one of the important pathophysiological mechanisms in the development of cardiovascular diseases [24]. A recent study suggested that hypoxia of the esophageal mucosa, which is caused by endothelial dysfunction, is an important factor in the development of GERD because it reduces the esophageal tissue resistance and causes dysfunction of the esophageal lower sphincter [23, 25].

A population-based cohort study conducted in Taiwan reported that the probability of developing CHD was increased 1.49-fold in GERD patients [4]. Recent studies have reported on the association between GERD and CAD, especially with regard to vasospastic angina [19, 26]. A study reported that 20% of vasospastic angina patients had a medical history of GERD, suggesting that patients with chest pain and a history of GERD may have vasospastic angina [19].

However, to the best of our knowledge, data on the association between GERD and coronary atherosclerosis are insufficient, and there have been few well-designed large-scale studies to date. In this study, coronary atherosclerosis was evaluated using the CACS. The CACS is a strong predictor for the development of CHD [27]. The CACS provides a direct, noninvasive estimation of the atherosclerotic plaque burden in the coronary arteries by using either electron beam CT or multi-slice CT [28]. The CACS is one of the established surrogate markers of atherosclerosis and has an appropriate prognostic value that reflects the presence and the severity of CAD [29, 30].

Our study showed that GERD and a high CACS are not causal but are associated with both diseases. Specifically, the more severe the GERD is, the higher the CACS. These results are consistent with the results of a previous study that indicated that GERD was associated with an increased risk of developing CHD [4]. Old age, male sex, hypertension, and hypercholesterolemia indicated an increased risk of a high CACS in GERD patients. Therefore, if a GERD patient with these risk factors complains of atypical chest pain, a cardiovascular examination should be performed. Interestingly, although smoking and obesity were risk factors for GERD and a high CACS, no significant associations were noted between these factors and a high CACS in GERD patients. Large scale systematic studies on the associations between these factors and the risk of CHD in GERD patients are needed in the future.

This study did not analyze the effects of proton pump inhibitors (PPIs). PPI use is the most effective medical treatment for GERD symptoms and erosive esophagitis. Maintenance PPI therapy should be administered to patients with persistent GERD symptoms upon discontinuation of PPIs [31]. There have been reports that long-term PPI treatment might be associated with adverse effects or complications, including kidney disease, *Clostridium difficile* infection, osteoporosis, and gastric cancer [1]. Recently, several studies have shown the associations between long-term PPI use and cardiovascular events [4, 32, 33]. However, another randomized controlled trial reported that no major safety concerns, including cardiac problems arose during 5–12 years of continuous PPI therapy [34]. More data are needed to draw conclusions about the association between PPIs and CHD.

Calcium channel blockers (CCBs) and nitrates are risk factors for the development of GERD. These drugs reduce lower esophageal sphincter pressure, impair esophageal clearance, and decrease the amplitude of esophageal contractions [35]. Unfortunately, detailed information on antihypertensive drugs was not collected in this study, so it was not able to evaluate the effect of CCB and nitrate on GERD.

The strength of this study is that most of the subjects were asymptomatic or had mild symptoms. Given that this institution mainly performs health checkups, most of the people who visit this institution are asymptomatic or have only mild symptoms. Therefore, the results of this study might be applicable to the general population.

There are some points to note in this study. First, GERD was diagnosed by endoscopic evaluation when mucosal breaks were present at the EG junction. We included MCLs, such as Z-line blurring or focal hyperemic changes, in the GERD group and compared them with the normal control group. According to a nationwide multicenter prospective study in Korea, MCLs have risk factors similar to those of GERD and are highly related to upper gastrointestinal symptoms [9]. Thus, MCLs can be considered early endoscopic findings of GERD. Another study reported that the frequency of pathologic acid reflux with non-erosive reflux disease (NERD) was higher in patients with MCLs than in patients without such changes [36]. On the other hand, another report suggests that most of the endoscopic findings indicating minimal changes were not associated with GERD [37]. One of the reasons for the differences in the results of these studies may be the high interobserver variation in the diagnosis of MCLs.

Second, many of the patients with NERD would have been classified as having MCLs, but it is possible that some NERD patients with normal endoscopic findings were included in the normal control group, which may have affected the study results.

Third, since *H*. *pylori* infection may affect the incidence of GERD and coronary atherosclerosis [38, 39], it would be better if *H*. *pylori* infection was included in the logistic regression analysis. In this study, however, data on *H*. *pylori* infection were not collected because most of the subjects did not proceed with the *H*. *pylori* test.

In conclusion, although the presence of a high CACS did not increase the risk of GERD or vice versa, the proportion of high CACS was significantly higher in subjects with severe GERD. Age, male sex, hypertension, and hypercholesterolemia were risk factors for a high CACS (≥100) in patients with GERD. Therefore, it might be helpful to test for coronary atherosclerosis using the CACS in GERD patients with these risk factors.

## Supporting information

S1 File Patients’ questionnaire. (DOCX)

## References

[1] Maret-Ouda J, Markar SR, Lagergren J. Gastroesophageal Reflux Disease: A Review. JAMA. 2020;324(24):2536–47. Epub 2020/12/23. doi: 10.1001/jama.2020.21360 . 33351048

[2] Camilleri M, Dubois D, Coulie B, Jones M, Kahrilas PJ, Rentz AM, et al. Prevalence and socioeconomic impact of upper gastrointestinal disorders in the United States: results of the US Upper Gastrointestinal Study. Clin Gastroenterol Hepatol. 2005;3(6):543–52. Epub 2005/06/14. doi: 10.1016/s1542-3565(05)00153-9 . 15952096

[3] Hunt R, Armstrong D, Katelaris P, Afihene M, Bane A, Bhatia S, et al. World Gastroenterology Organisation Global Guidelines: GERD Global Perspective on Gastroesophageal Reflux Disease. J Clin Gastroenterol. 2017;51(6):467–78. Epub 2017/06/08. doi: 10.1097/MCG.0000000000000854 . 28591069

[4] Chen CH, Lin CL, Kao CH. Association between gastroesophageal reflux disease and coronary heart disease: A nationwide population-based analysis. Medicine (Baltimore). 2016;95(27):e4089. doi: 10.1097/MD.0000000000004089 ; PubMed Central PMCID: PMC5058831. 27399102 PMC5058831

[5] World Health Organization. Regional Office for the Western P. The Asia-Pacific perspective: redefining obesity and its treatment: Sydney: Health Communications Australia; 2000 2000.

[6] Armstrong D, Bennett JR, Blum AL, Dent J, De Dombal FT, Galmiche JP, et al. The endoscopic assessment of esophagitis: a progress report on observer agreement. Gastroenterology. 1996;111(1):85–92. Epub 1996/07/01. doi: 10.1053/gast.1996.v111.pm8698230 . 8698230

[7] Lundell LR, Dent J, Bennett JR, Blum AL, Armstrong D, Galmiche JP, et al. Endoscopic assessment of oesophagitis: clinical and functional correlates and further validation of the Los Angeles classification. Gut. 1999;45(2):172–80. Epub 1999/07/14. doi: 10.1136/gut.45.2.172 ; PubMed Central PMCID: PMC1727604. 10403727 PMC1727604

[8] Nakamura T, Shirakawa K, Masuyama H, Sugaya H, Hiraishi H, Terano A. Minimal change oesophagitis: a disease with characteristic differences to erosive oesophagitis. Aliment Pharmacol Ther. 2005;21 Suppl 2:19–26. Epub 2005/06/10. doi: 10.1111/j.1365-2036.2005.02469.x . 15943842

[9] Lee JH, Kim N, Chung IK, Jo YJ, Seo GS, Kim SW, et al. Clinical significance of minimal change lesions of the esophagus in a healthy Korean population: a nationwide multi-center prospective study. J Gastroenterol Hepatol. 2008;23(7 Pt 1):1153–7. Epub 2008/01/22. doi: 10.1111/j.1440-1746.2008.05299.x . 18205773

[10] Agatston AS, Janowitz WR, Hildner FJ, Zusmer NR, Viamonte M, Detrano R. Quantification of Coronary-Artery Calcium Using Ultrafast Computed-Tomography. Journal of the American College of Cardiology. 1990;15(4):827–32. doi: 10.1016/0735-1097(90)90282-t PubMed PMID: WOS:A1990CV36700012. 2407762

[11] Keelan PC, Bielak LF, Ashai K, Jamjoum LS, Denktas AE, Rumberger JA, et al. Long-term prognostic value of coronary calcification detected by electron-beam computed tomography in patients undergoing coronary angiography. Circulation. 2001;104(4):412–7. doi: 10.1161/hc2901.093112 . 11468202

[12] Hanifehpour R, Motevalli M, Ghanaati H, Shahriari M, Aliyari Ghasabeh M. Diagnostic Accuracy of Coronary Calcium Score Less than 100 in Excluding Coronary Artery Disease. Iran J Radiol. 2016;13(2):e16705. Epub 20160320. doi: 10.5812/iranjradiol.16705 ; PubMed Central PMCID: PMC5035795. 27679688 PMC5035795

[13] Franklin BA, Brubaker P, Harber MP, Lavie CJ, Myers J, Kaminsky LA. The Journal of Cardiopulmonary Rehabilitation and Prevention at 40 yr and Its Role in Promoting Preventive Cardiology: Part 2. J Cardiopulm Rehabil Prev. 2020;40(4):209–14. doi: 10.1097/HCR.0000000000000523 . 32604250

[14] Craven MA, Waterfall WE. The esophagus as a source of non-cardiac chest pain. Can Fam Physician. 1988;34:663–8. Epub 1988/03/01. ; PubMed Central PMCID: PMC2219036. 21253154 PMC2219036

[15] Bortolotti M, Marzocchi A, Bacchelli S, Esposti AD, Sarti P, Brunelli F, et al. The esophagus as a possible cause of chest pain in patients with and without angina pectoris. Hepatogastroenterology. 1990;37(3):316–8. Epub 1990/06/01. . 2373462

[16] Chauhan A, Mullins PA, Taylor G, Petch MC, Schofield PM. Cardioesophageal reflex: a mechanism for "linked angina" in patients with angiographically proven coronary artery disease. J Am Coll Cardiol. 1996;27(7):1621–8. doi: 10.1016/0735-1097(96)00041-1 . 8636546

[17] Johansson S, Wallander MA, Ruigomez A, Garcia Rodriguez LA. Is there any association between myocardial infarction, gastro-oesophageal reflux disease and acid-suppressing drugs? Aliment Pharmacol Ther. 2003;18(10):973–8. Epub 2003/11/18. doi: 10.1046/j.1365-2036.2003.01798.x . 14616162

[18] Dobrzycki S, Baniukiewicz A, Korecki J, Bachorzewska-Gajewska H, Prokopczuk P, Musial WJ, et al. Does gastro-esophageal reflux provoke the myocardial ischemia in patients with CAD? Int J Cardiol. 2005;104(1):67–72. Epub 2005/09/03. doi: 10.1016/j.ijcard.2004.10.018 . 16137512

[19] Teragawa H, Oshita C, Ueda T. History of gastroesophageal reflux disease in patients with suspected coronary artery disease. Heart Vessels. 2019;34(10):1631–8. Epub 2019/04/18. doi: 10.1007/s00380-019-01413-1 . 30993440

[20] Gesualdo M, Scicchitano P, Carbonara S, Ricci G, Principi M, Ierardi E, et al. The association between cardiac and gastrointestinal disorders: causal or casual link? J Cardiovasc Med (Hagerstown). 2016;17(5):330–8. doi: 10.2459/JCM.0000000000000351 . 26702598

[21] Lei WY, Wang JH, Wen SH, Yi CH, Hung JS, Liu TT, et al. Risk of acute myocardial infarction in patients with gastroesophageal reflux disease: A nationwide population-based study. PLoS One. 2017;12(3):e0173899. Epub 2017/03/21. doi: 10.1371/journal.pone.0173899 ; PubMed Central PMCID: PMC5358801. 28319162 PMC5358801

[22] Castell DO. Chest pain of undetermined origin: overview of pathophysiology. Am J Med. 1992;92(5A):2S–4S. Epub 1992/05/27. doi: 10.1016/0002-9343(92)80049-6 1595759

[23] Oparin A, Vnukova A. The Role of Endothelial Dysfunction in the Mechanism of Gastroesophageal Reflux Disease Development in Patients with Ischemic Heart Disease. Acta Clin Croat. 2017;56(4):635–9. Epub 2018/03/30. doi: 10.20471/acc.2017.56.04.08 . 29590716

[24] Schafer A, Bauersachs J. Endothelial dysfunction, impaired endogenous platelet inhibition and platelet activation in diabetes and atherosclerosis. Curr Vasc Pharmacol. 2008;6(1):52–60. Epub 2008/01/29. doi: 10.2174/157016108783331295 . 18220940

[25] Khomenko L, Vnukova A, Dvoiashkina Y. Features of Endothelial Dysfunction in Elderly Persons with Coronary Heart Disease and Concomitant Gastroesophageal Reflux Disease. Georgian Med News. 2019;(287):78–82. Epub 2019/04/09. . 30958293

[26] Sueda S. General internists of experience suspected variant angina as gastroesophageal reflux diseases in two cases: Heart burn may be related to coronary spasm. J Cardiol Cases. 2020;21(3):93–6. Epub 2020/03/11. doi: 10.1016/j.jccase.2019.10.008 ; PubMed Central PMCID: PMC7054655. 32153681 PMC7054655

[27] Detrano R, Guerci AD, Carr JJ, Bild DE, Burke G, Folsom AR, et al. Coronary calcium as a predictor of coronary events in four racial or ethnic groups. N Engl J Med. 2008;358(13):1336–45. Epub 2008/03/28. doi: 10.1056/NEJMoa072100 . 18367736

[28] Nucifora G, Bax JJ, van Werkhoven JM, Boogers MJ, Schuijf JD. Coronary artery calcium scoring in cardiovascular risk assessment. Cardiovasc Ther. 2011;29(6):e43–53. Epub 2010/06/18. doi: 10.1111/j.1755-5922.2010.00172.x . 20553289

[29] Moradi M, Nouri S, Nourozi A, Golbidi D. Prognostic Value of Coronary Artery Calcium Score for Determination of Presence and Severity of Coronary Artery Disease. Pol J Radiol. 2017;82:165–9. Epub 2017/04/11. doi: 10.12659/PJR.900643 ; PubMed Central PMCID: PMC5378275. 28392854 PMC5378275

[30] Pathakota SR, Durgaprasad R, Velam V, Ay L, Kasala L. Correlation of coronary artery calcium score and carotid artery intima-media thickness with severity of coronary artery disease. J Cardiovasc Thorac Res. 2020;12(2):78–83. Epub 2020/07/07. doi: 10.34172/jcvtr.2020.14 ; PubMed Central PMCID: PMC7321008. 32626546 PMC7321008

[31] Katz PO, Gerson LB, Vela MF. Guidelines for the diagnosis and management of gastroesophageal reflux disease. Am J Gastroenterol. 2013;108(3):308–28; quiz 29. Epub 2013/02/20. doi: 10.1038/ajg.2012.444 . 23419381

[32] Sun S, Cui Z, Zhou M, Li R, Li H, Zhang S, et al. Proton pump inhibitor monotherapy and the risk of cardiovascular events in patients with gastro-esophageal reflux disease: a meta-analysis. Neurogastroenterol Motil. 2017;29(2). Epub 2016/09/01. doi: 10.1111/nmo.12926 . 27577963

[33] Sehested TSG, Gerds TA, Fosbol EL, Hansen PW, Charlot MG, Carlson N, et al. Long-term use of proton pump inhibitors, dose-response relationship and associated risk of ischemic stroke and myocardial infarction. J Intern Med. 2018;283(3):268–81. Epub 2017/10/13. doi: 10.1111/joim.12698 . 29024109

[34] Attwood SE, Ell C, Galmiche JP, Fiocca R, Hatlebakk JG, Hasselgren B, et al. Long-term safety of proton pump inhibitor therapy assessed under controlled, randomised clinical trial conditions: data from the SOPRAN and LOTUS studies. Aliment Pharmacol Ther. 2015;41(11):1162–74. Epub 2015/04/11. doi: 10.1111/apt.13194 . 25858519

[35] Mungan Z, Pinarbasi Simsek B. Which drugs are risk factors for the development of gastroesophageal reflux disease? Turk J Gastroenterol. 2017;28(Suppl 1):S38–S43. doi: 10.5152/tjg.2017.11 . 29199166

[36] Joh T, Miwa H, Higuchi K, Shimatani T, Manabe N, Adachi K, et al. Validity of endoscopic classification of nonerosive reflux disease. J Gastroenterol. 2007;42(6):444–9. Epub 2007/08/03. doi: 10.1007/s00535-007-2022-3 . 17671758

[37] Kim JH, Park H, Lee YC, group Ms. Is minimal change esophagitis really part of the spectrum of endoscopic findings of gastroesophageal reflux disease? A prospective, multicenter study. Endoscopy. 2011;43(3):190–5. Epub 2011/03/03. doi: 10.1055/s-0030-1256101 . 21365512

[38] Boltin D, Niv Y, Schutte K, Schulz C. Review: Helicobacter pylori and non-malignant upper gastrointestinal diseases. Helicobacter. 2019;24 Suppl 1:e12637. doi: 10.1111/hel.12637 . 31486237

[39] Sun J, Rangan P, Bhat SS, Liu L. A Meta-Analysis of the Association between Helicobacter pylori Infection and Risk of Coronary Heart Disease from Published Prospective Studies. Helicobacter. 2016;21(1):11–23. Epub 20150522. doi: 10.1111/hel.12234 . 25997465

