# Supplementary material for: Association between gastroesophageal reflux disease and coronary atherosclerosis
Source: PLoS One. 2022 May 20;17(5):e0267053. doi: 10.1371/journal.pone.0267053 (PMC9122211; doi:10.1371/journal.pone.0267053)
Supplement: S1 File — (DOCX) [file pone.0267053.s001.docx]

**Patients’ Questionnaire**

1. Do you have below diseases? (multiple answers are possible)

| Diagnosis | Yes | Medication | Remark |
| --- | --- | --- | --- |
| Hypertension | O | O |  |
| Diabetes | O | O |  |
| Hyperlipidemia | O | O |  |
|  |  |  |  |
| O Don’t have any above diseases | | | |

2. Are you taking or injecting any medication for more than 2 months in purpose of treatment? (multiple answers are possible)

| O Don’t take any medication | |
| --- | --- |
|  | |
| O Antiplatelet (Aspirin, Clopidogrel) | O Anticoagulant |
| O Antihypertensive agent | O Antihyperlipidemic agent |
| O Antianginal agent | O Antidiabetic agent |

3. Have any of your family members (parents, siblings or children) been diagnosed or died with following diseases?

| O Angina/ Myocardiac Infarction |
| --- |

4. Have you ever smoked in your life?

| O Never smoked |
| --- |
| O Smoked in the past but not now |
| O Current smoking |

4-1. If smoked before, but currently quit (Former smoker)

| How long has it been since you quit smoking? ____ year(s) |
| --- |
| Before quitting, how many years did you smoke? ____ year(s) |
| Before quitting, how much did you smoke per day? ______ cigarette(s) |

4-2. If currently smoking (Current smoker)

| How long have you been smoking? ____ year(s) |
| --- |
| How much do you smoke per day? ______ cigarette(s) |

5. How often do you drink alcohol?

| O Not applicable | O 2 days/week | O 5 days/week |
| --- | --- | --- |
| O Less than once a month | O 3 days/week | O 6 days/week |
| O 2~4 days/month | O 4 days/week | O 7 days/week |

6. How many do you usually drink at a time?

| O 1~2 glass(es) |
| --- |
| O 3~4 glasses |
| O 5~6 glasses |
| O 7~9 glasses |
| O 10 glasses and more |

1. 아래의 질환이 있다고 진단받은 적이 있다면 해당항목에 표시해 주십시오.

| 질환명 | 진단력 | 투약중 | 비고 |
| --- | --- | --- | --- |
| 고혈압 | O | O |  |
| 당뇨병 | O | O |  |
| 고지혈증 | O | O |  |
|  | | | |
| O 상기 질환이 없다. | | | |

2. 아래의 약물 중 1주일에 2번 이상 지속적으로 복용하는 경우 표시해 주십시오.

| O 아래의 약을 복용하지 않는다 | |
| --- | --- |
|  | |
| O 항혈소판제 (아스피린) | O 항응고제 (와파린, 쿠마딘) |
| O 고혈압약 | O 고지혈증약 |
| O 협심증약 | O 당뇨약 |

3. 부모, 형제, 자매, 자녀 중 아래의 질환을 앓았거나 그로 인해 사망한 경우가 있습니까?

| O 협심증, 심근경색 |
| --- |

4. 담배를 피우십니까?

| O 피운적이 없다 |
| --- |
| O 이전에 피웠으나 끊었다 |
| O 현재도 피우고 있다 |

4-1. 과거에는 흡연하였으나 현재는 끊었다면

| 금연하신지는 몇 년 되셨습니까? ____ 년 |
| --- |
| 금연 전까지 담배를 몇 년이나 피우셨습니까? ____ 년 |
| 금연하시기 전 평균 하루 흡연량은 몇 개비였습니까? ______ 개비 |

4-2. 현재도 흡연을 하신다면

| 몇 년 째 담배를 피우고 계십니까? ____ 년 |
| --- |
| 하루 평균 흡연량은 얼마입니까? ______ 개비 |

5. 술을 얼마나 자주 마십니까?

| O 해당사항 없음 | O 일주일에 2일 | O 일주일에 5일 |
| --- | --- | --- |
| O 한 달에 1일 이하 | O 일주일에 3일 | O 일주일에 6일 |
| O 한 달에 2~4일 | O 일주일에 4일 | O 일주일에 7일 |

6. 술을 드실 때 하루에 보통 몇 잔씩 마십니까?

| O 1~2 잔 |
| --- |
| O 3~4 잔 |
| O 5~6 잔 |
| O 7~9 잔 |
| O 10 잔 이상 |
